# Quantifying Contextual Interference and Its Effect on Skill Transfer in Skilled Youth Tennis Players

**DOI:** 10.3389/fpsyg.2017.01931

**Published:** 2017-11-03

**Authors:** Tim Buszard, Machar Reid, Lyndon Krause, Stephanie Kovalchik, Damian Farrow

**Affiliations:** ^1^Institute of Sport, Exercise and Active Living, Victoria University, Footscray, VIC, Australia; ^2^Game Insight Group, Tennis Australia, Melbourne, VIC, Australia; ^3^Skill Acquisition, Australian Institute of Sport, Bruce, ACT, Australia

**Keywords:** sport science, motor skill acquisition, contextual interference effect, expertise, tennis, skill transfer

## Abstract

The contextual interference effect is a well-established motor learning phenomenon. Most of the contextual interference effect literature has addressed simple skills, while less is known about the role of contextual interference in complex sport skill practice, particularly with respect to skilled performers. The purpose of this study was to assess contextual interference when practicing the tennis serve. Study 1 evaluated tennis serve practice of nine skilled youth tennis players using a novel statistical metric developed specifically to measure between-skill and within-skill variability as sources of contextual interference. This metric highlighted that skilled tennis players typically engaged in serve practice that featured low contextual interference. In Study 2, 16 skilled youth tennis players participated in 10 practice sessions that aimed to improve serving “down the T.” Participants were stratified into a low contextual interference practice group (Low CI) and a moderate contextual interference practice group (Moderate CI). Pre- and post-tests were conducted 1 week before and 1 week after the practice period. Testing involved a skill test, which assessed serving performance in a closed setting, and a transfer test, which assessed serving performance in a match-play setting. No significant contextual interference differences were observed with respect to practice performance. However, analysis of pre- and post-test serve performance revealed significant Group × Time interactions. The Moderate CI group showed no change in serving performance (service displacement from the T) from pre- to post-test in the skill test, but did display improvements in the transfer test. Conversely, the Low CI group improved serving performance (service displacement from the T) in the skill test but not the transfer test. Results suggest that the typical contextual interference effect is less clear when practicing a complex motor skill, at least with the tennis serve skill evaluated here. We encourage researchers and applied sport scientists to use our statistical metric to measure contextual interference.

## Introduction

The contextual interference effect is a well-established motor learning phenomenon. It refers to the interference that is experienced when practicing multiple skills, or variations of a skill, within a single practice session (Shea and Morgan, 1979). High contextual interference emerges when multiple skills are practiced one after the other, whereas low contextual interference transpires when one skill is repeatedly practiced before progressing to another skill. The most intriguing aspect of the contextual interference effect is the inverse relationship that is apparent between performance during practice and performance during skill-retention and skill-transfer tests. Low contextual interference practice typically produces better performance during practice, whereas high contextual interference practice leads to better performance during retention and transfer tests (for reviews, see Magill and Hall, 1990; Brady, 1998, 2004; Barreiros et al., 2007).

Support for high contextual interference practice has mostly come from laboratory-based experiments that consider the learning of simple motor skills. Less emphatic are the findings in applied environments where complex motor skills are practiced (Farrow and Buszard, 2017). By way of example, a meta-analysis on contextual interference found that effect sizes were much larger for laboratory-based experiments than applied experiments (i.e., experiments that assess the acquisition of sport skills; Brady, 1998). Barreiros et al. (2007) revealed a positive contextual interference effect with regards to skill retention in only 11 of 27 studies in applied settings^1^. Collectively, these results illustrate the possible incongruence between the learning observed in laboratory versus applied settings. This is a concern for practitioners, such as sports coaches, who are responsible for the organization of practice when working in the field.

One possible cause of the reduced efficacy of high contextual interference in applied settings may be due to the relative difficulty of the skill being practiced. A number of researchers have suggested that a certain level of skill is required to reap the benefits of practice that features high contextual interference (e.g., Magill and Hall, 1990; Hebert et al., 1996; Farrow and Maschette, 1997; Guadagnoli and Lee, 2004). Support for this argument comes from observations that the learning of complex skills, including golf putting, basketball shooting and throwing, were enhanced when contextual interference gradually increased as skill developed (Porter and Magill, 2010; Hodges et al., 2011; Saemi et al., 2012). Unfortunately, few studies have assessed the contextual interference effect in applied settings with highly skilled performers, therein making it difficult to conclude whether high contextual interference practice is beneficial when the skill level of a performer is more advanced (for exceptions, see Hall et al., 1994; Ollis et al., 2005).

One key reason for this lack of research on skilled performers is likely due to the difficulty associated with controlling experimental conditions in naturalistic environments. Skilled performers, such as athletes, are less inclined to modify practice for the purpose of an experiment if the benefits are not clear. Researchers therefore need to develop novel approaches to objectively assess practice via observation. If contextual interference could be quantified during practice, then researchers and practitioners could objectively compare a variety of practice approaches without interfering with the daily practice of the performers. To this point, there has been limited focus in developing a metric to quantify contextual interference during practice.

Another complication for applied studies of contextual interference is a lack of appropriate skill transfer measures. Measures of skill transfer arguably provide the best indicator of learning, as the purpose of practice is to apply the learned skill in other environments (Shewokis and Snow, 1997). In sport, skill transfer refers to the ability to perform the learned skill in competition. Remarkably, only one study has assessed the transfer of skill to the competition setting when examining the contextual interference effect (Cheong et al., 2016). Instead, researchers have tended to measure skill transfer by assessing the ability to perform the skill in a new context, but the new context is never actual competition.

To enhance our understanding of the contextual interference effect in applied settings, we designed two studies that (a) assessed a complex applied skill with skilled performers, (b) developed a metric to quantify the amount of contextual interference experienced in practice, and (c) examined skill transfer in a competition setting. The first study entailed the development of a metric to quantify contextual interference. We used this metric to assess serving practice of highly skilled youth tennis players during training. This metric also informed our interpretation of the relationship between contextual interference and performance during practice. The second study compared the effect of low and moderate contextual interference practice on learning a specific tennis serve (serving “down the T”). Critically, we assessed whether contextual interference influenced skill transfer to a competition setting.

## Study 1

**FIGURE 1:**
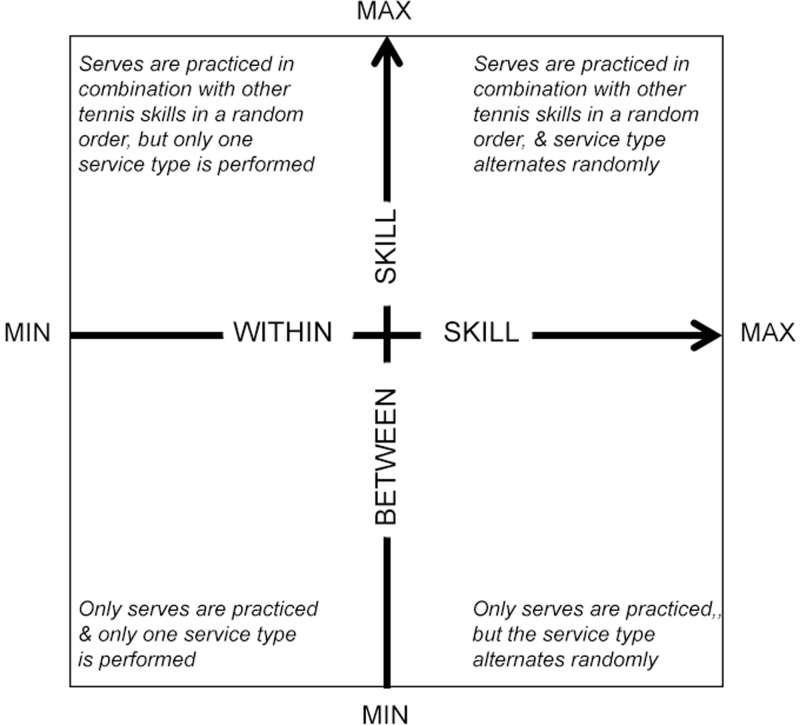
Between-skill and within-skill variability interactions that define the continuum of contextual interference in a tennis application for service practice. The minimum and maximum labels refer to inter-trial variability during practice. Adapted from Reid et al. (2007).

Traditionally contextual interference has been defined as either low or high. However, this dichotomous characterisation of practice might not accurately capture the amount of contextual interference to which performers are exposed. Moreover, the degree of contextual interference applied by a coach in a practical setting is usually the product of two variables, which for the purpose of this paper will be defined as *between-skill variability* and *within-skill variability*. Between-skill variability refers to the switching of skills during practice (e.g., practicing a tennis serve followed by a forehand), whereas within-skill variability refers to the discernible variation in the execution of the same skill (e.g., practicing a T serve followed by a wide serve; see **Figure 1**). Between-skill variability has traditionally been referred to as variations between motor programs, whereas within-skill variability refers to variations within a motor program (Magill and Hall, 1990). Terms such as “blocked,” “serial” and “random” practice have traditionally been used to describe the magnitude of between-skill variability, whilst within-skill variability has been described as “constant” and “variable” practice. To date, there is no way of objectively describing the combined effect of between-skill and within-skill variability to quantify the degree of contextual interference in naturalistic settings.

Previous observations of sports practice suggests that athletes and coaches habitually structure practice that is low in contextual interference (Williams and Hodges, 2005). We suspect that this tendency is driven by: (i) the immediacy of visible changes in motor performance caused by blocked practice that are misconstrued as a permanent change in that performance (i.e., the coach incorrectly concludes learning has occurred); and (ii) practice reflecting the way in which coaches practiced as players (Reid et al., 2007). Lower contextual interference practice is also thought to be easier to implement and conducive to remedial technical practice or confidence building (e.g., Abraham and Collins, 2011). However, given that lower contextual interference practice might produce diminished learning, over-habituation to this type of practice might be sub-optimal. The aim of Study 1 was therefore to establish the practice habits of skilled youth tennis players through the lens of contextual interference and with service skill in mind. We also aimed to identify whether the relationship between contextual interference and practice performance in a naturalistic setting is consistent with experimental findings, with higher contextual interference resulting in poorer performance.

### Materials and Methods

#### Participants

Nine highly skilled youth tennis players that were training at the National Tennis Academy (Melbourne) participated in the study. The players included 7 males aged 14 to 22 years (mean = 17.1 years, *SD* = 3.0 years) and 2 females aged 15 and 16 years. All players held top 16 national age group rankings (*n* = 5, top 5; and *n* = 4, top 6–16) and an Australian or open ranking that ranged from 24 to 618, with seven players ranked in the top 150. The two players that were ranked outside of the top 150 were the two youngest players (aged 14 years). Across the nine players there were seven coaches – one coach for the two female players (F1–F2), one coach for two male players (M4–M5), and five coaches for the remaining five players. All participants provided written informed assent and their parents/guardians provided written informed consent. The protocol was approved by the Victoria University Human Research Committee.

#### Procedure

Training for each player was notated for the duration of one training block, which consisted of 3–4 weeks of daily practice scheduled between tournaments. Training was assessed by a research assistant who was independent to the study’s aims. Assessment occurred via video replay using the SportsCode sports analytics software (SportsCode, Jounieh, Lebanon). Given that this study specifically examined the serve, only drills and activities that included serving were analyzed. In addition, match-play and point-play during practice were excluded from the analysis given we were only interested in the characteristics of practice drills that targeted serving. Point-play was defined by activities where points were played but the typical tennis scoring rules (i.e., games and sets) were not followed. We acknowledge that the exclusion of match-play and point-play creates a bias in our assessment of tennis practice. However, we were solely interested in drill-based activities, as the order of practice during drills is typically dictated by the coach and/or player (as opposed to during match-play or point-play whereby the rules of tennis demand constant skill switching).

Six-to-twelve practice sessions were filmed per player (median = 8 sessions per player), equating to 77 practice sessions in total. A practice session was defined as an on-court coach-led training session whereby the primary focus was the performance of tennis skills (as opposed to physical conditioning). A single session could include a number of drills of which any number may or may not have focused on serving. A drill was defined as a distinct task within a session. When the task shifted focus (e.g., changing from serves only to forehands only), or a prolonged rest period took place (e.g., sitting down for a few minutes), the drill was deemed to have finished. Of the 77 practice sessions analyzed, 39 sessions included serving drills (median = 4 sessions per player). To ensure that we captured enough data per drill, only drills that featured 10 serves or more were included in the analysis. In total, 54 drills across 39 sessions were analyzed, which corresponded to a median of 6 drills per player (note: these numbers represent the cumulative total of drills. They do not represent the number of unique drills).

#### Assessment of Practice Data

The aim of the practice assessment was to accurately illustrate between-skill and within-skill variability. Three performance measures were extracted from the recordings.

##### Tennis shot type

Categorized as a serve, serve return, forehand, backhand, volley or an overhead smash. Each type of shot was mutually exclusive from one another (i.e., returns and volleys were not considered forehands or backhands. Forehands and backhands were reserved for groundstrokes (i.e., all shots that were played after the ball had bounced). The total number of shots analyzed was 1885 (serves = 1551, forehands = 200, backhands = 106, volleys = 26, smash = 2).

##### Service side

Defined as the side of the court that the serve was played from (i.e., the Deuce side or Advantage side).

##### Service placement

**FIGURE 2:**
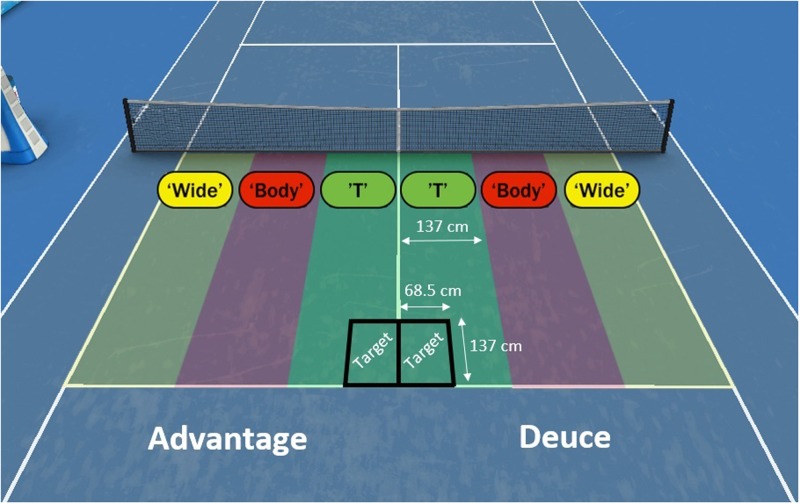
An illustration of the performance measures *service side* (Deuce and Advantage) and *service placement* (Wide, Body, T) in Study 1, and the target used during practice in Study 2. [Image created using HawkEye^TM^ proprietary data visualization software. HawkEye^TM^ granted us permission to publish this image].

Defined as the direction that the serve was hit. This was categorized as either a T serve (i.e., a serve that was directed down the middle of the court), a body serve (i.e., a serve that was directed to the middle of the service box), or a wide serve (i.e., a serve that was directed to the widest part of the service box) in accordance with the dimensions in **Figure 2**.

Serving accuracy was also assessed. Serves were recorded as either successful (i.e., landed in the service box) or a fault (i.e., landed outside the service box). The percentage of successful serves was calculated relative to the total number of serves.

One drill per player was reassessed by a second researcher to assess inter-rater reliability. This equated to 364 shots being recoded, which represented 19% of the total data. Intra-class coefficients (ICC) were calculated for each performance measure (i.e., total number of serves, total number of forehands, total number of backhands, etc.). ICC varied between 0.85 and 0.99 across each performance measure, indicating moderate-to-high inter-rater reliability.

#### Statistical Analysis

Estimates of between-skill and within-skill variability were obtained from the autocorrelation estimate from a first-order autoregression model. This model assumes that the expected skill at time *t* is a function of the skill performed immediately beforehand *(t-1)* and the measure of autocorrelation of interest, which will be denoted as ρ, is between the current event (i.e., the current shot) and the previous event (i.e., the previous shot) in a sequence. The autocorrelation ρ takes a value between -1 and 1. The magnitude of ρ is measured by its absolute value |ρ| and a higher magnitude reflects a more predictable and less variable sequence of skills, whereas a magnitude closer to 0 indicates a highly variable sequence. Since a strong negative or strong positive were equally reflective of the predictability of the sequence, we focus on the magnitude of the correlation, |ρ|, as a measure of the strength of a pattern of skills. This value was then subtracted from 1, so that higher values represented larger variability. Between-skill and within-skill variability was therefore calculated from the estimated autocorrelation as 1 -|ρ|. By transforming the value so that a high score represents larger variability, as opposed to the opposite, the value is aligned with **Figure 1**.

Separate autoregressive models were fit for the (a) sequence of shot types (between-skill variability) and (b) sequence of service side and *service placement* (within-skill variability) for each training session. This allowed us to obtain separate estimates for the between-skill and within-skill variability for each drill. The outcome of the between-skill model was an indicator of whether a serve was performed (1 if a serve was performed, 0 otherwise). Hence, between-skill variability was measured by the sequence of the serve versus other skills. For the within-skill model, we subset the analyses to all serves that were performed and the outcome (sequence of events) was a 6-category indicator describing service side (Ad/Deuce) and *service placement* (Wide/Body/T) used. This measured the sequence of switches in service location patterns. To isolate the contribution to variation of service side and *service placement*, we fit separate autoregressive models with one model defining the serve sequence only by service side and the other model defined only by serve *service placement*. We then estimated the Spearmen correlation between the within-skill variability estimates with each of these models to the 6-category service pattern model to determine whether service side or *service placement* contributed more to the overall variation in serving patterns.

The autoregressive models were fit using the gls function in R (R Core Team, 2014; see **Supplementary Material** for the R code used to calculate between-skill and within-skill variability). Means and standard deviations for between-skill and within-skill variability are reported as well as the correlation (*r*) between these variables and serving accuracy. Statistical significance was accepted at *p* < 0.05.

### Results

**FIGURE 3:**
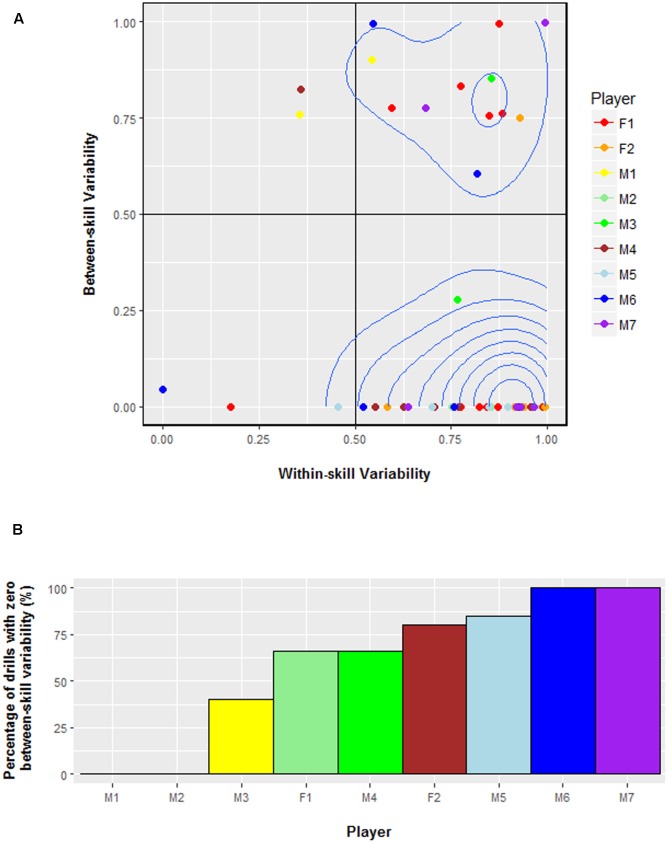
**(A)** Between-skill and within-skill variability values for every serving drill by each player in Study 1. A 2-D density plot was added to the figure to highlight the most common between-skill and within-skill variability values. A value of 0 represents minimum variability whereas 1 represents maximum variability. **(B)** The percentage of drills that featured zero between-skill variability for each player in Study 1. The number of drills assessed per player were: M1 = 2, M2 = 8, M3 = 2, M4 = 7, M5 = 4, M6 = 5, M7 = 6, F1 = 15, F2 = 5. Hence, the two players that did not experience any drills with zero between-skill variability had the least number of serving drills assessed.

**Figure 3A** illustrates that a large portion of serving practice featured zero between-skill variability, inferring that the serve was often practiced in isolation from other skills. Six of nine players practiced the serve with zero between-skill variability in more than 50% of drills (**Figure 3B**). The mean between-skill variability score across all players was 0.22 (*SD* = 0.37). Conversely, the mean within-skill variability was considerably higher (*M* = 0.77, *SD* = 0.22). Within-skill variability was more highly correlated to *service placement* (*r* = 0.97) than to service side (*r* = 0.41). Not surprisingly, it was observed that the player’s coach influenced the results, as the players’ coached by the same person recorded similar values. These included players M4–M5 and F1–F2. This occurred because these players often practiced in the same session.

With regards to serving accuracy, weak and non-significant correlations were observed between the percentage of serves that landed in the service box and between-skill variability (*r* = 0.15, *p* = 0.26) and within-skill variability (*r* = 0.16, *p* = 0.24). The mean percentage of serves that landed in the service box across all players was 65% (*SD* = 14%).

### Discussion

The metric developed to quantify contextual interference provided an objective assessment of between-skill and within-skill variability in a naturalistic tennis setting. Two observations were gleaned from this analysis. First, between-skill variability was low across seven of the nine players. This highlights that players tended to practice serving in large blocks without incorporating other skills. This resembles observations in other sports, whereby practice often features minimal contextual interference (Williams and Hodges, 2005). Second, within-skill variability was much higher than between-skill variability, and it appeared that this was due to players more frequently changing the direction of their serves rather than changing the type of shot played. We suspect that this was largely due to the fact that player and coaches tend to set goals for improving one specific skill at a time (e.g., the sole aim of the drill might be to improve the serve), and manipulating within-skill variability still allows players the opportunity to achieve the task goal. However, a limitation of this study was that we did not capture the exact aims of each session from each player and coach.

Notably, no significant correlations were found between serving accuracy and either between-skill or within-skill variability. Typically low contextual interference practice leads to better performance during practice compared to high contextual interference practice. We propose that our finding can be explained by the complexity of the serving skill. In other words, because the serve requires a high level of coordination, a larger number of repetitions may be required to meet our definition of serving success (accuracy) here. There are three other potential explanations for this observed effect. First, the definition of accuracy was crude and it could be that when assessed against more specific target areas, players perform differently. Second, given that different types of serves are hit with different speed and precision (Whiteside and Reid, 2017), it might be expected that an analysis that is able to differentiate between those serve types (i.e., first serves rather than a combination of first and second serves as was undertaken in this study) might reveal a different result. Third, it is possible that the players were not focused on serving accuracy, but instead were focused on their movement pattern. Nonetheless, this result is consistent with other studies that have examined applied skills (e.g., Jones and French, 2007; Cheong et al., 2012; Cheong and Lay, 2013; Rouhollahi et al., 2014).

If performance during practice does not differ between higher and lower contextual interference practice for complex skills, however, the key question then becomes whether learning differences – skill retention and skill transfer – emerge between the two practice conditions, as is the case in typical contextual interference studies. In other words, do learning differences emerge between practice schedules of differing contextual interference, even if there are no differences during practice? If skill transfer differences do emerge in favor of higher contextual interference, then this would indicate that current serving practice habits (as illustrated in Study 1) might be producing sub-optimal results. Study 2 aimed to answer this question.

## Study 2

A training study was conducted to demonstrate the effect of contextual interference on learning to serve “down the T” by skilled youth tennis players. Contextual interference was manipulated by altering between-skill variability in practice. We chose to alter between-skill variability as this was the factor that the contextual interference effect was initially based on. We restricted the skill of interest to serves “down the T” as we wanted to maximize the number of trials for one skill (as opposed to practicing three types of serves).

Based on the observations in Study 1, we speculated that contextual interference would not influence performance during practice. However, if contextual interference did not affect performance in practice, then there was also reason to believe that contextual interference would have minimal influence on skill learning. Indeed, many previous applied studies that failed to find differences in practice performance between high and low contextual interference protocols also revealed no differences in skill learning (French et al., 1990; Jones and French, 2007; Cheong et al., 2012; Cheong and Lay, 2013; Rouhollahi et al., 2014). These studies, however, examined participants with low levels of skill relative to the task. The limited study of skilled performers reveals that differences in skill retention still exist despite minimal differences in performance during practice (Hall et al., 1994). Hence, we expected to find differences in skill retention regardless of whether contextual interference influenced performance during practice.

A novel feature of this study was the assessment of performance during competition as a means to measure skill transfer. If contextual interference does influence the learning of skilled performers, this should be evident in measures of skill transfer. In the one previous study that used competition to measure skill transfer, contextual interference had minimal effect on the performance of hockey skills during practice, but did positively influence performance during game-play (Cheong et al., 2016). We therefore hypothesized that contextual interference would influence skill transfer results.

### Materials and Methods

#### Participants

Sixteen skilled youth tennis players aged 11 to 13 years participated in the study. These participants did not participate in Study 1. The participants comprised of eight males (mean = 12.1 years, *SD* = 0.4 years) and eight females (mean = 12.1 years, *SD* = 0.9 years) and were ranked in the top 50 players for their age group. Participants were recruited from a training squad that was considered to involve the best players in the state for their age group. All participants provided written informed assent and their parents/guardians provided written informed consent. The protocol was approved by the Victoria University Human Research Committee.

Participants were allocated into two groups – a low contextual interference group, referred to as Low CI; and a moderate contextual interference group, referred to as Moderate CI. Participants were allocated into these groups based on serving ability. Serving ability of each player was assessed on a 3-point scale by an expert tennis coach with high performance qualifications^2^. A higher score represented better serving ability. The mean serving score for each group was: Low CI = 1.8 (*SD* = 0.8); Moderate CI = 1.9 (*SD* = 0.8).

#### Experimental Design

This study used a randomized design to test the effect of contextual interference. Players were randomly stratified into a Low CI group (*n* = 8) or a Moderate CI group (*n* = 8). The Low CI group practiced under low contextual interference conditions that would be typically referred to as *blocked* practice. Blocked practice involves practicing skills in isolation from one another. The Moderate CI group practiced under moderate contextual interference conditions that would typically be referred to as *serial* practice. *Serial* practice involves the constant switching of skills but in a predictable order. Within-skill variability remained constant for both groups.

The experimental design comprised of a pre-test, 10 practice sessions and a post-test. The aim during practice was to improve “first serves down the T.” Pre- and post-tests consisted of a skill test and a transfer test. The skill test assessed serving performance in a closed setting, while the transfer test assessed serving performance in a match-play setting.

#### Practice Protocol

Participants attended 10 sessions of practice over a 7 week period. The practice sessions were part of the participants’ regular training sessions, with the serving practice occurring during the first 30 min of every session. Participants were instructed to “practice first serves down the T,” aiming at target zones that were placed adjacent to the corners of the deuce and advantage service box’s and the T (137 cm × 68.5 cm). Hence, the target box was positioned in the center corner of each service box and furthest from the net. The number of serves that landed inside the target zone was recorded for each session. Furthermore, in an attempt to better replicate match-play conditions, participants from the same group attempted to return their opponents’ serves deep and crosscourt (a common tennis return tactic). We considered the replication of a performance environment, which includes opponents responding to serves, to be important for maximizing skill transfer.

A total of 40 serves and 40 groundstrokes were practiced during the first 30 min of each session. The groundstrokes were included as a means to modify the degree of contextual interference. These shots involved one player feeding the ball (underarm) to start a rally. We chose not to analyze the groundstrokes as we were only interested in improving serving skill. The Low CI group practiced 40 consecutive serves followed by 40 consecutive groundstrokes. Given that serving occurs in two different locations during match-play (deuce side of the court and the advantage side of court), the first 20 serves for the Low CI group were on the deuce side and the second 20 serves were on the advantage side. The order of serving and groundstroke practice (i.e., which skill was practiced first) was counterbalanced across the 10 sessions. The Moderate CI group also practiced 40 serves and 40 groundstrokes; however, this was divided into 10 blocks consisting of 4 consecutive serves followed by 4 consecutive groundstrokes. We based the Moderate CI group’s practice on a *serial* practice schedule as this was practically more feasible (i.e., asking players to switch every 4 shots was far easier to control than asking players to switch after a randomly allocated number). The Moderate CI group also practiced the serve on the deuce and advantage sides of the court, with rotation also occurring after the first 20 serves were complete. Thus, the only difference between the two groups was the scheduling of groundstroke practice amongst the serving practice. The Low CI group was considered to have low contextual interference given that 40 serves were practiced without any interference from other skills, whereas the Moderate CI group was thought to have moderate contextual interference given that the groundstrokes practiced frequently interfered with the serving practice. Applying our between-skill variability scale (Study 1), the Moderate CI session design had a score of 0.46, whereas Low CI practice schedule had a score of 0.

#### Testing Protocol

A skill test and a transfer test was administered pre and post the practice period.

##### Skill test

Participants were asked to serve 40 balls, with the first 20 hit from the deuce side of the court and the second 20 from the advantage side. Participants were instructed to: “imagine that it is a first serve and your aim is to serve as close as possible to the T.” No instruction was provided about service velocity as we wanted to observe typical first serves, rather than simply the fastest serves (although they may be related). There was no returner present for this task as the intent was for the player to solely focus on hitting their serve accurately down the T.

##### Transfer test

Participants played two matches against other participants across 2 days. The opponents for each match were determined by the head coach as part of a competition that the players were involved in. Because this was part of regular competition, participants were given no instruction related to their serve.

#### Performance Measures and Analysis

##### Practice performance

###### Serves hitting target

This was defined as the number of serves that landed in the target box (located in a position that characterizes a T serve) during each practice session. An aggregate score for each practice session was used for analysis.

##### Skill test

###### Serves-in

This was defined as the number of first serves that landed in the service box (successful serves) and is expressed as a percentage of the total number of serves.

###### Service displacement from the T

This represents the distance in centimeters between the ball’s landing location and the T. This was measured using the computer software Siliconcoach (Siliconcoach, Dunedin, New Zealand), which includes a function that measures distance in a video, provided a standard reference measurement is given. To achieve this, a digital video camera was set-up behind the court (4 m past the baseline and 4 m above the ground), with the camera angle perpendicular to the service line. The distance from the T to the singles line (along the service line) was used to calibrate the two-dimensional space (4.115 m). Only serves that landed in the service box (i.e., successful serves) were assessed. Unsuccessful serves were not assessed as these are invalid shots in a tennis match.

###### Service velocity

This was expressed in kilometers per hour (kph) and was measured using a Stalker Sport 2 Radar Gun (Applied Concepts, Inc./Stalker Radar, Richardson, TX, United States). Only serves that landed in the service box (i.e., successful serves) were recorded.

##### Transfer test

###### T Serves-in

The same definition as used for the skill test was applied. However, only serves that landed in an area defined as a T serve were analyzed. A T serve was defined as any ball that landed within one-third of the center line (also known at the “T”). This equated to 1.37 m either side of the center line.

###### Service displacement from the T

The same definition as used for the skill test was applied. However, only T serves were analyzed (i.e., balls that landed within 1.37 m of the center line).

###### First service down the T

This was defined as the number of first serves that were directed to the T location (i.e., balls that landed within 1.37 m of the center line) and is expressed as a percentage relative to the total number of first serves.

#### Statistical Tests

Mixed modeling was used to estimate the serve performance characteristics of each group and time period, including during practice. In these models, the correlation induced by the irregular number of repeated measures from each subject was captured by a random effect for subject. Included fixed effects were Group (Low CI and Moderate CI), Time (pre-test and post-test), and their interaction. When the outcome was service displacement from the T or service velocity, normal residual error was used. For the binary outcome of a serves-in, T serves-in, and first service down the T, a generalized linear mixed model was used with a logistic link function. For the evaluation of the number of serves hitting a target during practice, a regression model appropriate for count data was needed. We used the Poisson regression model as it is a common model of count data. The regression analysis of this model focuses on the rate of events out of a known number of trials. To ensure the positivity of the rate, we use the standard log link (Cameron and Trivedi, 1998).

Statistical inferences about group differences were based on a likelihood ratio test of the full model with the effect in question (i.e., the interaction between Group and Time) against the model without the effect in question. The likelihood ratio test was performed with a Chi-square distribution using the appropriate degrees of freedom for the comparisons being made. Assessments about the magnitude of effects between groups were based on linear contrasts of the model fixed effects and their 95% confidence intervals using the Holm method to adjust for multiple comparisons. Likewise, for all performance measures, we used the Holm method to adjust *p-*values for four comparisons: difference between the two groups during pre-test and post-test, and the difference between pre- and post-test within each group. For the assessment of practice performance, only one *p-*value was calculated. Statistical significance was accepted at *p* < 0.05. There were four counts of missing data: one participant from the Low CI group was absent for post-skill test and the post-transfer test, another participant from the Low CI group was absent for the post-skill test, while one participant from the Moderate CI group was absent for the pre-transfer test. All analyses were performed in the R language (R Core Team, 2014) and the *lme4* package (Bates et al., 2015) was used for the mixed modeling.

### Results

#### Practice Performance

Both groups performed similarly during practice. Across the 10 practice sessions, the Low CI group hit the target on 36% (95% CI [30%, 43%]) of the trials, while the Moderate CI group hit the target on 37% (95% CI [31%, 45%]) of the trials (*p* = 0.80).

#### Skill Test

##### Serves-in

No differences were evident between the Low CI and Moderate CI groups at pre-test (*p* = 1.0) or post-test (*p* = 1.0), nor did either group display a significant change from pre- to post-test (*p* = 1.0). Furthermore, a likelihood ratio test showed that the interaction between Group and Time had no significant effect on serves-in [χ^2^(1) = 0.17, *p* = 0.68]. The descriptive statistics reported in **Table 1**.

**Table 1:** The estimated mean and 95% confidence intervals for each performance measure assessed at pre- and post-test for the skill test in Study 2.

Performance measure	Time	Low CI	Moderate CI	Δ
Serves-in (%)	Pre-test	31.7% (26.1%, 37.5%)	29.9% (24.6%, 36.7%)	-1.6% (-9.7%, 9.2%,)
	Post-test	35.2% (29.1%, 41.6%)	31.2% (25.7%, 37.1%)	-3.3% (-10.7%, 6.9%)
	Δ	3.6% (-41.6%, 15.6%)	1.3% (-7.1%, 11.9%)	

Service displacement from the T (cm)	Pre-test	108.9 (93.6, 124.1)	82.6 (67.3, 97.9)	-26.3 (-55.2, 2.7)
	Post-test	68.2 (52.0, 84.3)	79.4 (63.7, 95.2)	11.3 (-18.9, 41.5)
	Δ	-40.7 (-63.5, -17.9)	-3.2 (-25.6, 19.3)	

Service velocity (kph)	Pre-test	129.0 (123.7, 134.3)	133.2 (128.0, 138.5)	4.3 (-6.0, 14.5)
	Post-test	131.3 (126.1, 136.7)	133.0 (127.7, 138.3)	1.7 (-8.6, 11.9)
	Δ	2.4 (1.0, 3.8)	-0.2 (-1.5, 1.0)	


*Δ denotes the estimated difference between pre- and post-test for each group or the difference between each practice group at pre-test and post-test.*

##### Service displacement from the T

There were no significant differences between the groups at pre-test (*p* = 0.06) or post-test (*p* = 0.63). However, a likelihood ratio test revealed that the interaction in our model (Group × Time) had a significant effect on distance [χ^2^(1) = 9.40, *p* = 0.002]. This interaction is evident based on the estimated means and 95% confident intervals in **Table 1**, with the Low CI group improving performance from pre- to post-test more than the Moderate CI group by 37.7 cm (95% CI [13.8, 62.3]). Linear contrasts highlighted that the improvement from pre- to post-test was significant for the Low CI group (*p* < 0.0001) but not for the Moderate CI group (*p* = 0.72).

##### Service velocity

No differences were apparent at pre-test (*p* = 0.72) or post-test (*p* = 1.0) between the two groups, but an interaction (Group × Time) revealed a significant effect on speed [χ^2^(1) = 12.98, *p* = 0.0003]. A significant change from pre- to post-test was evident for the Low CI group (*p* < 0.0001) but not the Moderate CI group (*p* = 1.0). The estimated means and confidence intervals (**Table 1**) suggest that the Low CI group increased their service velocity by 2.4 kph (95% CI [1.0, 3.8]), while the Moderate CI group maintained the same serve speed.

#### Transfer Test

##### T Serves-in

There were no differences between the two groups at pre-test (*p* = 1.0) or post-test (*p* = 1.0), nor did either group display a significant change from pre- to post-test (*p* = 1.0). The likelihood ratio test revealed no significant effect between our interaction (Group × Time) and serves-in [χ^2^(1) = 0.34, *p* = 0.56]. The descriptive statistics for each group are reported in **Table 2**.

**Table 2:** The estimated mean and 95% confidence intervals for each performance measure assessed at pre- and post-test for the transfer test in Study 2.

Performance measure	Time	Low CI	Moderate CI	Δ
T serves-in (%)	Pre-test	56.7% (51.8%, 61.3%)	56.0% (50.8%, 61.3%)	-0.5% (-7.8%, 10.6%)
	Post-test	52.5% (46.8%, 58.4%)	55.9% (49.3%, 60.8%)	2.8% (-7.1%, 18.3%)
	Δ	-4.0% (-8.4%, 6.4%)	-1.0% (-8.2%, 10.1%)	

Service displacement from the T (cm)				
	Pre-test	74.0 (65.0, 83.0)	80.6 (65.0, 83.0)	6.6 (-11.4, 24.6)
	Post-test	89.6 (77.5, 101.8)	70.1 (59.4, 80.8)	-19.5 (-41.0, 2.0)
	Δ	15.6 (-2.9, 34.2)	-10.5 (-28.0, 7.0)	

First service down the T (%)	Pre-test	30.8% (26.5%, 35.7%)	34.8% (29.9%, 40.1%)	4.7% (-5.1%, 17.3%)
	Post-test	27.7% (23.0%, 33.2%)	33.4% (28.1%, 39.2%)	7.2% (4.5%, 23.7%)
	Δ	-3.1% (-9.2%, 6.1%)	-1.4% (-9.2%, 8.7%)	


*Δ denotes the estimated difference between pre- and post-test for each group or the difference between each practice group at pre-test and post-test.*

##### Service displacement from the T

There were no significant differences between the groups at pre-test (*p* = 0.29) or post-test (*p* = 0.07). However, there was a significant interaction effect (Group × Time) for distance [χ^2^(1) = 7.40, *p* = 0.007]. **Table 2** shows that the post-test performance of the Low CI group deteriorated, whereas the Moderate CI group improved. The Moderate CI group hit their serves closer to the T during match-play by 26.1 cm (95% CI [7.4, 45.1]) more than the Low CI group. Although, it must be noted that the change from pre-test to post-test was not statistically significant for either group (Low CI, *p* = 0.08; Moderate CI, *p* = 0.23).

##### First service down the T

There were no differences between the two groups at pre-test (*p* = 0.25) or post-test (*p* = 0.56), nor did either group display a significant change from pre- to post-test (Low CI, *p* = 0.34; Moderate CI, *p* = 0.75). The interaction between Group and Time was found to have a negligible effect on directing first serves down the T [χ^2^(1) = 0.15, *p* = 0.70]. The descriptive statistics are reported in **Table 2**.

### Discussion

Study 2 indicated that the manipulation of contextual interference during serving practice influenced the transference of skill to competition by skilled youth tennis players. This occurred despite contextual interference having no influence on performance during practice. Two differences emerged between the Low CI and Moderate CI groups in pre-/post-test measures of performance and skill transfer. First, whilst manipulating contextual interference had no effect on serving success (i.e., serving to the service box), it did influence the ability to serve closer (i.e., with more accuracy) to the T. Specifically, the Low CI group displayed improvements in the skill test, but a decline in performance in the transfer test. Conversely, the Moderate CI group showed no change in the skill test, but did improve the ability to serve closer to the T during the transfer test. These results conform to the observation by Cheong et al. (2016) that skill tests might not capture the important differences between practice groups. For complex skills, the contextual interference effect might only become evident when the skill is assessed in transfer conditions representative of the competitive setting in naturalistic environments. Second, the Low CI group, as opposed to the Moderate CI group, also served faster in the skill test following the practice period. Service velocity was not measured during the transfer test, so we cannot comment on the transferability of service velocity.

The results of this study need to be interpreted in light of some limitations. First, we cannot be certain that the assessment of serving during the transfer test (match-play) captured the player’s intent (in other words, the players may or may not have been intending to serve down the T). For instance, it is possible that some serves deemed to be T serves may have been intended for another part of the service box (i.e., aimed wide or body). Likewise, some serves that were intended to be T serves may have landed in a location outside of the T area. Future studies could ask players to report the intention of each serve prior to serving to minimize this error. We opted for a more naturalistic and representative performance environment, with match-play occurring free of interruptions. A second limitation was that match-play pairings, during pre- and post-testing, were not the same owing to a coach-led competition that the players were engaged in.

We should also be mindful of the reason the Likelihood ratio test revealed that the interaction in the mixed model (Group × Time) had a significant influence on serving closer to the T during the transfer test. There were no differences between the groups at pre-test, nor at post-test, and neither group displayed a significant change from pre- to post-test. An argument could therefore be postulated that there were no differences between the groups. However, the significant interaction indicates that each group changed from pre- to post-test differently. Indeed, the Low CI group’s performance declined whereas the Moderate CI group’s performance improved. Hence, neither group displayed a significant change in performance from pre-test to post-test according to the 0.05 *p-*value threshold, but the interaction was significant. To put the data into perspective, the Low CI group improved service displacement from the T by the diameter of almost six tennis balls in the skill test, while the Moderate CI group improved serving distance to the T by almost two tennis balls during the transfer test (Note: the standard tennis ball diameter is 6.86 cm).

Additionally, the lack of clear difference between the groups might have been due to insufficient practice and/or a small sample size. In other words, if a contextual interference effect does exist when learning complex motor skills, it might require more than 10 practice sessions and/or a greater sample size for it to be clearly identified statistically. Unfortunately, both of these issues represent major challenges for researchers in this field, as skilled performers are often reluctant to alter their training program to that of an experiment.

We should also consider the influence of individual differences when assessing the contextual interference effect. According to the Challenge-Point framework (Guadagnoli and Lee, 2004), learning is heightened when contextual interference is matched to the performer’s skill level for a given task. A practical example of this is the Win-Shift-Lose-Stay methodology (Simon et al., 2008). This concept suggests that contextual interference should only increase when the performer experiences success. *Post hoc* exploration of our data revealed that 7 participants showed large improvements in serving closer to the T during the transfer test (>30 cm closer to the T than pre-test) – 5 of these participants were a part of the Moderate CI group and the other 2 participants were a part of the Low CI group. This offers support for the argument that individual-specific levels of contextual interference might be required to enhance skill transfer.

## General Discussion

Four outcomes materialized from the two studies. First, we demonstrated that it is possible to measure the amount of contextual interference in practice in a naturalistic setting. Second, using this metric, it was evident that skilled youth tennis players typically engaged in serving practice that featured low contextual interference. Specifically, between-skill variability was low, meaning that players tended to practice the serve in isolation from other skills. This is problematic if such practice delivers minimal transfer to competition. Third, contextual interference appeared not to influence practice performance, which upon first glance contradicts previous contextual interference findings. Typically, greater contextual interference suppresses practice performance but results in superior learning (Magill and Hall, 1990; Brady, 1998, 2004; Barreiros et al., 2007). Indeed, we suspect that the classical differences observed between lower and higher contextual interference groups during practice are overridden when the motor skill is relatively complex. Fourth, despite a lack of expected difference between the Moderate CI group and the Low CI group during practice, Study 2 revealed an interaction between practice group and performance change from pre- to post-test. Specifically, the Moderate CI group displayed greater improvements in the transfer test relative to the Low CI group. This suggests that practice that is higher in contextual interference is advantageous for skilled performers refining complex motor skills in applied environments. Interestingly, the Low CI practice group displayed greater improvements in the skill test.

Whilst the results of both studies do not reinforce the classic contextual interference effect, the data supports higher contextual interference practice as a means to enhance skill transfer to competition for the tennis serve. In Study 2, contextual interference was manipulated by increasing between-skill variability. Our results therefore suggests that greater switching between skills is beneficial for skilled performers when learning complex skills. According to traditional (information processing) theories of contextual interference, task switching enhances learning due to constant reconstruction of the motor plan or elaborate processing of the motor plan. The *forgetting-reconstruction hypothesis* claims that high contextual interference causes the performer to constantly forget task-specific information between practice trials, therein necessitating the (re)construction of an action plan for every trial (Lee and Magill, 1983, 1985). Consequently, the performer is thought to become more adept in developing action plans, which subsequently facilitates greater skill retention (e.g., Kim et al., 2015). The *elaboration hypothesis* proposed a similar account; however, rather than ‘forgetting’ information between trials during higher contextual interference practice, proponents argue that the performer engages in more elaborate processing to represent the motor skill in long-term memory (e.g., Shea and Morgan, 1979; Shea and Zimny, 1983, 1988). These traditional theories are supported by neuropsychological measures, with a number of studies highlighting neural correlate differences between higher and lower contextual interference practice schedules (for reviews, see Lage et al., 2015; Wright et al., 2016). However, whilst these accounts explain the enhanced skill transfer for the Moderate CI group in Study 2, they do not explain why the results for the two groups were reversed during the skill test.

An alternate explanation of our data can be derived from a much simpler account – practice specificity. Practice specificity suggests that learning is enhanced when practice more closely replicates the demands of the intended performance environment (e.g., Lee, 1988). Certainly practice specificity explains the results of the Low CI group on the skill test. The Low CI group practiced 40 consecutive serves every session. This was the same as the skill test, which also featured 40 consecutive serves. Hence, the Low CI group improved their ability to serve accurately when there was 40 consecutive serves. Conversely, the Moderate CI group only performed four consecutive serves during practice, which was more closely aligned with competition (hence, the transfer test). Consistent with the specificity notion, the Moderate CI group improved serving performance in the transfer test. Additionally, the Low CI group displayed greater improvement in the skill test compared to the Moderate CI group’s improvement in the transfer test. This difference might have occurred since the Low CI group’s practice schedule was the same as the skill test whereas the Moderate CI group’s practice schedule was not identical to the transfer test. Thus, the amount of improvement might have related to how closely practice mirrored the test.

This explanation resembles the conclusion by Russell and Newell (2007), who tested this hypothesis with simple motor skills (touching targets on a computer screen) in a well-controlled laboratory setting. In their study, participants who were exposed to a random practice schedule displayed superior retention of skill when tested under random, but not blocked, conditions. The authors concluded: “If future performance will occur in an open environment, in which the task varies from trial to trial, then [random] practice is essential…. if future performance will occur in a closed environment in which the task remains constant then [blocked] practice is at least as beneficial as [random] practice” (pp. 326).

However, we must be careful drawing this conclusion. Hall et al. (1994) reported that a random practice schedule enhanced skill retention of a baseball batting task for skilled players, irrespective of whether the retention test followed a blocked or a random schedule. Likewise, Hebert et al. (1996) found that lower contextual interference during practice was more beneficial for less skilled performers, regardless of the testing schedule. Thus, a strong recommendation with regard to practice specificity in applied contexts cannot be made.

Investigations of contextual interference have produced inconsistent findings within applied settings (e.g., Hall et al., 1994; Ollis et al., 2005; Jones and French, 2007; Cheong et al., 2012). To reconcile the contextual interference debate, we encourage researchers to adopt transfer tests that measure skills in their true environment (e.g., competition). Although this represents a challenge, the rise of technology and data analytics has made it possible to measure performance with minimal error during game-play (e.g., Hawkeye Technology; Whiteside and Reid, 2017). Moreover, we hope applied sports scientists working with athletes will use our contextual interference metric to assess the relationship between practice schedule and performance change. For example, during training blocks whereby athletes aim to improve one or more skills, the sport scientist can assess contextual interference during each drill of each session. Contextual interference can then be tested as a potential predictor variable of performance change across the training block.

## Conclusion

A novel feature of this study was the development of a statistical metric to quantify contextual interference during practice. We recommend researchers interested in contextual interference in sport to use this metric as it will facilitate more accurate comparisons across studies. It is also worth considering how this metric can be combined with other factors that are known to influence motor performance and learning, such as frequency of feedback. Significantly, we hope the metric will be used to assess practice in naturalistic settings, particularly practice by skilled performers who are typically more reluctant to subject their practice time to an experimental condition.

The results of Study 2 suggested that greater between-skill variability, which increased contextual interference, was beneficial for transferring serving skill to tennis competition. If between-skill variability does facilitate skill transfer, then it is possible that many skilled youth tennis players are engaging in sub-optimal practice (based on the observations in Study 1). Moreover, since our observations of tennis practice reflect similar observations of theory-practice divisions in other sports (Williams and Hodges, 2005), we suspect that athletes across many sports are engaging in practice that could be considered redundant if there is a lack of transfer to competition.

## Ethics Statement

This study was carried out in accordance with the recommendations of the National Statement on Ethical Conduct in Human Research (2007). All participants gave written informed assent and written informed consent was provided by their parents or guardians in accordance with the National Statement. The protocol was approved by the Victoria University Human Research Ethics Committee.

## Author Contributions

TB led the study. His involvement included designing the study, collecting the data, analyzing the data, and writing the manuscript. MR and DF made a significant contribution to designing the study, data interpretation and writing the manuscript. LK played a major role during data collection, and also contributed to data interpretation and manuscript writing. SK made a significant contribution with the data analysis, data interpretation, and writing the manuscript.

## Conflict of Interest Statement

The authors declare that the research was conducted in the absence of any commercial or financial relationships that could be construed as a potential conflict of interest.

## Footnotes

1 Barreiros et al. (2007) defined an applied setting as when a common task was used (e.g., sport skills) in their natural setting (e.g., physical education and training environments), coupled with a normal amount and distribution of practice.

2 The high performance coach education course is the highest qualification for a tennis coach in Australia.

## Supplementary Material

**DATA SHEET 1 |** Sample data that can be used for trialling the R code used to measure between-skill and within-skill variability in Study 1.
